# Coccydynia Improved by Percutaneous Discectomy

**DOI:** 10.7759/cureus.74564

**Published:** 2024-11-27

**Authors:** Reon Kobayashi, Ichiro Okano, Asae Taketomi, Eiko Hara, Hitoshi Mera

**Affiliations:** 1 Anesthesiology, Showa University School of Medicine, Tokyo, JPN; 2 Orthopaedic Surgery, Showa University School of Medicine, Tokyo, JPN

**Keywords:** coccyx, discectomy, intervertebral disc displacement, lumbar disc, minimally invasive lumbar decompression, nerve block, percutaneous, percutaneous disc decompression

## Abstract

Usually, coccydynia cases are caused by herniated discs, with lower back pain and sciatica as initial symptoms. However, whether lumbar disc herniation causes coccydynia without back pain remains unclear. We report a case of lumbar disc herniation diagnosed as the underlying cause of coccydynia by discoblock.

A woman in her mid-20s was treated for coccydynia experienced during sitting, for several years. There was no coccyx tenderness upon palpation. As the location of the pain could not be identified, it was not possible to perform a block at the site of the pain. Magnetic resonance imaging showed a herniated L5/S1 lumbar disc, without lower back pain and sciatica. Following discoblock, coccydynia was diagnosed as associated pain due to the herniated L5/S1 lumbar disc that was treated with percutaneous discectomy. After surgery, coccydynia was relieved while sitting; no medication was required.Discoblock was used to diagnose lumbar disc herniation as the cause of coccydynia. Percutaneous discectomy was effective for coccydynia without back pain, thus lumbar disc herniation should be considered as a differential diagnosis. Discoblock can be useful for differentiation.

## Introduction

Coccydynia is pain originating in the caudal region and can significantly diminish the patient's quality of life, necessitating appropriate pain management. Coccydynia etiologies include soft tissue abnormalities around the coccyx, pelvic floor muscle spasms, pain associated with lumbar spine pathology, lower sacral nerve root arachnoiditis, and trauma; however, a considerable number of cases remain idiopathic [1]. Whether lumbar disc herniation is a cause of coccydynia with an absence of back pain remains unclear.

Discoblock [2], which entails the injection of a low-dose local anesthetic into a disc followed by the patient assuming a posture that triggers pain, has been a complementary diagnostic tool for provocative discography of lumbar disc herniation [3].

Minimally invasive techniques, including decompression percutaneous discectomy, percutaneous coblation nucleation, and chemolysis, have been developed as alternatives to surgery, particularly for small disc herniations [4]. Although various electrothermal and radiofrequency catheters can reach the internal annulus [5], percutaneous discectomy using the L’DISQ (U&I Corporation, Uijeongbu-si, Republic of Korea) has demonstrated potential efficacy in managing lower back pain in patients with lumbar disc herniation [6] because it has the advantage of being able to better decompress the posterior lateral annulus nuclear tissue using navigation technology. However, comprehensive criteria for surgical indications and prognosis have yet to be fully elucidated, thus further specific evidence is required. Notably, there are no reports regarding L’DISQ use for coccydynia management.

We report a case of lumbar disc herniation diagnosed as the underlying cause of coccydynia by discoblock.

## Case presentation

**Figure 1:**
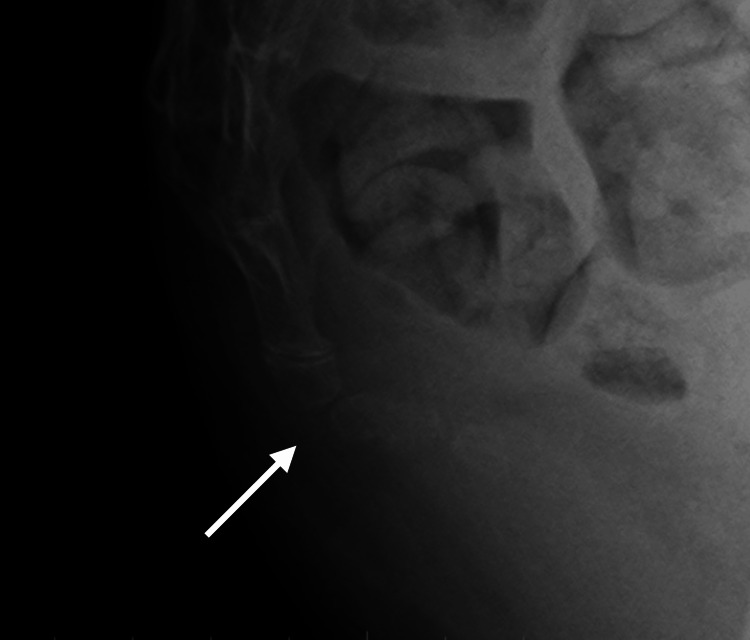
Coccy X-ray imaging The white arrow indicates suspected sacroiliac joint dislocation, although there is no tenderness.

A woman in her mid-20s (height 156.7 cm, weight 44.4 kg) presented with the chief complaint of coccydynia during prolonged sitting. Injury or trauma to the area was not indicated. She had received conservative treatment with tramadol 150 mg/day and acetaminophen 1300 mg/day for several years. X-ray images of the tailbone suggested the possibility of sacroiliac joint dislocation, but physical examination revealed no reproducible pain on palpation of the tailbone (Figure 1).

**Figure 2:**
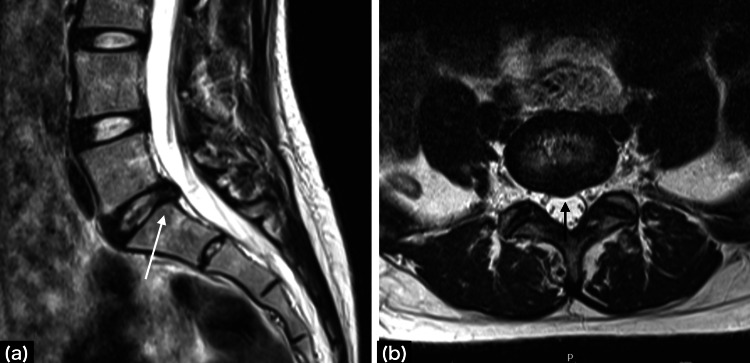
Magnetic resonance imaging T2-weighted images (a) Sagittal view; a posteriorly protruding disc at high L5/S1 is observed. The white arrows indicate mild HIZs, (b) Axial view of L5/S1; a posterior midline protruding herniated disc is observed. The black arrows indicate mild HIZs. HIZs: high-intensity zones

There was also no evidence of fractures or trauma. Lumbar spine X-ray showed L4 spondylolysis and magnetic resonance imaging (MRI) showed a lumbar disc herniation at the L5/S1 level (Figure 2).

**Figure 3:**
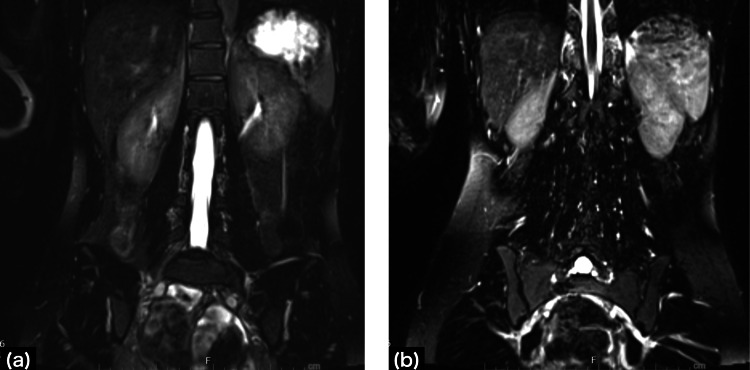
Magnetic resonance imaging T2-weighted images (a) Coronal view of the bilateral L5 root nerves; no calcareous deposits compressing nerve roots, including L5/S1, (b) coronal view of the gluteus maximus muscle level; there is no entrapment of the sciatic nerve in the deep gluteal muscles or anatomic variation of the sciatic nerve.

The hernia was located in the midline and had mild high-intensity zones (HIZs). No obvious positional abnormalities were noted. In addition, all anatomical abnormalities, nerve root abnormalities, and deep gluteal muscle abnormalities that could cause coccydynia were ruled out by MRI (Figure 3).

There was no tenderness upon palpation of the coccyx and no back or leg pain symptoms on physical examination. The patient was referred to a pain clinic to discontinue medication because of her desire to have a baby. At the initial pain clinic visit, the patient reported pain from the lower sacrum to the coccyx. There was no accompanying lower back pain and neurological examination findings of the lower extremities were negative. Since there was no reproducible pain on palpation of the tailbone, the location of the pain could not be clearly identified, and it was not possible to perform a block in the area of pain. We discussed with the patient whether to perform a coccygeal block or an epidural block first. As a result, we were unable to obtain consent for a coccygeal block, so we decided to perform an epidural block, and an epidural block from L5/S1 was performed. The patient's tailbone pain was relieved for approximately 12 hours thereafter.

**Figure 4:**
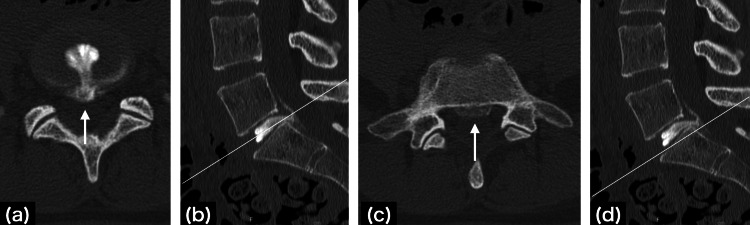
Post-discoblock computed tomography (a) Axial view at the level of (b), (b) midline sagittal view, (c) axial view at the level of (d), and (d) midline sagittal view. A lumbar disc herniation protruding centrally (white arrows) is observed showing migration both upward and downward and is a contained type that does not penetrate the posterior longitudinal ligament.

Since the coccydynia was temporarily alleviated with an epidural block, lumbar disc herniation was considered the cause of the pain. One week later, provocative discography and discoblock were performed simultaneously at the L5/S1 level. Following confirmation of proper needle placement within the disc, 2.0 ml of contrast medium was injected, followed by 2.0 ml of 1% mepivacaine. Injection was performed manually and patently as usual, and injection pressure was not increased to the maximum extent possible. Therefore, no reproducible pain was obtained. However, 30 minutes after discoblock, a notable reduction in coccydynia during sitting was achieved. Additionally, on the same day, post-discoblock computed tomography confirmed a contained-type disc herniation migrating up and down the midline (Figure 4).

Thus, the cause of the coccydynia was determined as discogenic pain associated with lumbar disc herniation, leading to the decision to proceed with a percutaneous discectomy.

One month after visiting the pain clinic, a percutaneous discectomy was performed. Prior to percutaneous discectomy, the numerical rating scale and EuroQol 5-dimensions 5-levels (EQ5D-5L) scores were 6 and 0.610, respectively, and pain level measured by the current perception threshold test using PainVision^®^ PS-2100 (Osachi Corporation, Okaya, Japan) was 738. EQ5D-5L is one of the most widely tested and applied patient-reported outcome measures in the world. It includes five important aspects of health (mobility, self-care, usual activities, pain/discomfort, anxiety/depression) and has five levels. In addition, the PainVision^®^ PS-2100 is used to quantitatively determine the intensity of pain as the “degree of pain” calculated from the current generation of pain perception, which is equivalent to the current generation of electrical threshold perception.

**Figure 5:**
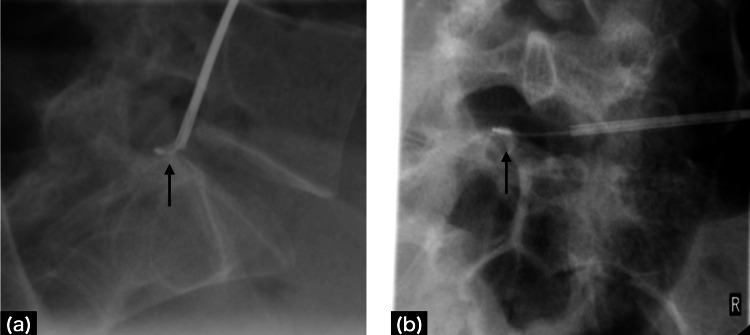
Percutaneous discectomy using L’DISQ (a) Lateral fluoroscopic view illustrating the wand tip (black arrow) touching the posterior longitudinal ligament within the herniation, (b) anteroposterior fluoroscopic view illustrating the wand tip (black arrow positioned in the center of the herniation)

The L’DISQ device was used. The patient was positioned left laterally, with support under the lower back to achieve a convex alignment. The puncture point was located 11.5 cm from the midline, and the lateral supine angle was adjusted to ensure that the lateral margin of the superior articular process was approximated for 3/5 of the vertebral body with the C-arm. The L’DISQ electrode was inserted through the lateral margin of the superior articular process. The wand was bent backward, ensuring that the needle tip did not protrude far from the posterior surface of the vertebral body in the lateral view and was located near the spinous process in the frontal view. Ablation was performed at the point where the tip of the wand was a few millimeters further back than at the location where it would not advance over the posterior longitudinal ligament. Throughout the ablation procedure, continuous monitoring using C-arm fluoroscopy ensured the accurate placement of the wand tip, which was continuously moved to maximize the contact area during ablation. Ablation was performed for 323 seconds (Figure 5).

**Figure 6:**
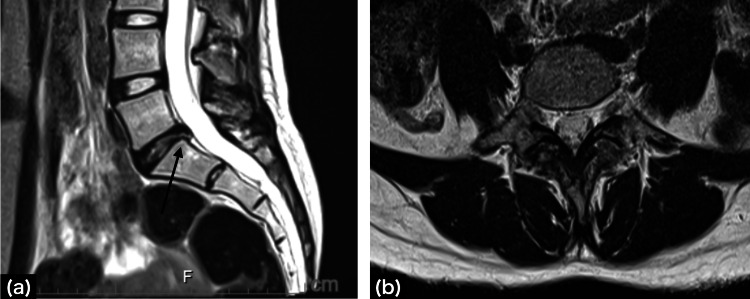
Six-month postoperative magnetic resonance T2-weighted image (a) Sagittal view; a posteriorly protruding disc at L5/S1 height is observed. The white arrows indicate mild HIZs. (b) Axial view of L5/S1; no obvious disc herniation is observed. HIZs: high-intensity zones

The day after the surgery, the patient reported a numeric rating scale (NRS) score of 7 for low back pain at the L5/S1 level. Consequently, L5/S1 intradiscal pulsed radiofrequency at 42 °C for 15 minutes was administered to address discogenic back pain. Two weeks postoperatively, the patient’s NRS score for coccydynia was 2, the EQ5D-5L score was 0.844, and the pain level assessed by PainVision^®^ was 226. At this point, the coccydynia improved, although a heavy dull ache at the L5/S1 level remained. At two months of follow-up, the NRS score for coccydynia was 3 and EQ5D-5L was 0.844. Therefore, the patient’s initial goal to terminate oral medication was achieved. At six months of follow-up, the NRS score for coccydynia was 3 and the EQ5D-5L score was 0.844. MRI showed a resolved midline hernia. The tailbone pain was considerably reduced (Figure 6).

## Discussion

Tailbone pain without back pain was diagnosed as lumbar disc herniation by disc block. Percutaneous discectomy was performed, and tailbone pain was relieved. Comparing the period before and after percutaneous discectomy, the NRS and EQ5D-5L scores changed from 6 to 3 and 0.610 to 0.844, respectively. Also, the patient was able to discontinue all oral medications including opioids.

There are many previous reports of tailbone pain arising from the lumbar spine, which has been regarded as associated pain from the lumbosacral disc [7,8]. However, in all these reports, low back pain has also been observed. The most common etiology of tailbone pain without low back pain is trauma. Non-traumatic tailbone pain can result from several causes, including obesity, female gender, degenerative joints, disc disease, hypermobility or hypomobility of the sacroiliac joint, and variations in tailbone morphology [9]. Very few patients have such a mixture of symptoms that it is impossible to distinguish tailbone pain syndrome from generalized low back pain syndrome, which is considered to be caused by local musculoskeletal abnormalities in the caudal region, with lumbosacral disc prolapse not being an important factor [7].

In this case, despite the observation of L5/S1 disc degeneration on MRI, there was no usual accompanying lower back or leg pain, and coccydynia was only observed while sitting, which is different from the findings of a previous report [7]. In addition, there was no trauma history. Sacral epidural blocks have historically been used for coccydynia management [10]; however, reports on the effectiveness of this technique remain inconclusive. Conversely, epidural injections have shown efficacy in treating intervertebral disc-related pain [3]. Given the temporary relief provided by the epidural block and considering that there was no reproducible pain on palpation of the coccyx, this patient’s condition was attributed to lumbar spine pathology, which led to the decision to perform provocative discography and discoblock.

The discoblock procedure confirmed that lumbar disc herniation was the underlying cause of coccydynia. Putzier et al. compared provocative discography and discoblock for idiopathic disc degeneration and demonstrated that the discoblock was an effective diagnostic tool for identifying pain due to lumbar disc degeneration [11]. Discoblock is also considered a more appropriate evaluation method for surgical decisions because it is independent of injection pressure and rupture type, can detect modic changes, and allows quantitative pain assessment.

Although the injection pressure was high, no reproducible pain was observed on discography. The patient only had pain in the sitting position when the lumbar spine was subjected to upper body weight, which strongly compresses the intervertebral discs. DePalma et al. reported nociceptor sensitization of intradiscal ruptures as a cause of discogenic pain [12]. The reason for the absence of reproducible pain with manual discography in this case may have been that the manual stimulation did not exceed the threshold of the nociceptors. The L5/S1 disc herniation compresses the sciatic plexus, and as the sciatic plexus provides muscular branches to various leg muscles, disc-stressing movements (e.g. sitting) may have caused disc-derived tailbone pain [13].

Sitting and other disc-straining movements may also cause compression of the sciatic plexus and tailbone pain through multiple mechanisms, including calcification compressing nerve roots [14], muscle entrapment in the deep gluteal muscles [15], external pressure on the sciatic nerve [16], and anatomical variations [17]. However, in the present case, these findings were all ruled out by MRI. 

Tailbone pain due to external pressure on the sciatic nerve is often caused by falls or prolonged sitting but this is unlikely, as there is no obvious history of falls, and the patient is often in a standing position at work.

Another unique aspect of this case is the use of percutaneous discectomy with L'DISQ for tailbone pain, which was effective. Percutaneous discectomy is a minimally invasive treatment for herniated discs, although there are no reports of its use for caudalgia. Lee et al. performed percutaneous discectomy with L'DISQ in 27 patients with neuropraxia due to a herniated disc; the average VAS score decreased from 7.08 before treatment to 1.84 at 24 weeks after the procedure [6]. In contrast, Calisaneller et al. reported that 14 patients (48.2%) had worse VAS scores at 6 months than at 24 hours postoperatively [18]. Cuellar et al. also evaluated MRI before and after nucleoplasty in 28 patients with lumbar disc herniation and the Pfirrmann scores revealed no detectable morphological disc improvement; 32% had progressive degeneration within 1 year after nucleoplasty, which was a period of examination greater than the rate expected due to natural progression, as noted by the authors [19]. However, these reports did not use the L'DISQ [18,19], and the efficacy and safety of this new minimally invasive technique have not yet been proven.

Kim et al. examined success factors for discogenic low back pain using the L’DISQ and reported that lack of migration, presence of HIZs, and unilaterality were success factors [20]. Regarding the absence of migration and presence of HIZs, the preoperative MRI of this case is consistent with that in the report of Kim et al. [20]; however, the combination treatment with L’DISQ may have been more effective for the tailbone pain because a disc pulse was added the day after surgery.

## Conclusions

Discoblock determined that the cause of coccydynia without back pain was lumbar disc herniation in this patient. Percutaneous discectomy using the L’DISQ was effective for treating coccydynia, and all oral medications, including opioids, were discontinued. When coccydynia that cannot be reproduced by palpation is observed, lumbar disc herniation should be considered a differential diagnosis, and discoblock can be useful for differentiation.

## References

[1] De Andrés J, Chave S (2003). Coccygodynia: a proposal for an algorithm for treatment. J Pain.

[2] Ohtori S, Kinoshita T, Yamashita M (2009). Results of surgery for discogenic low back pain: a randomized study using discography versus discoblock for diagnosis. Spine (Phila Pa 1976).

[3] Manchikanti L, Benyamin RM, Singh V (2013). An update of the systematic appraisal of the accuracy and utility of lumbar discography in chronic low back pain. Pain Physician.

[4] Guarnieri G, Vassallo P, Pezzullo MG (2009). A comparison of minimally invasive techniques in percutaneous treatment of lumbar herniated discs. A review. Neuroradiol J.

[5] Fasoli F, Gandini R, Scaggiante J, Bartolo M, Capobianco SV, Cerone G (2022). Minimally-invasive percutaneous treatments for low back pain and leg pain: a randomized controlled study of thermal disc decompression versus mechanical percutaneous disc decompression. Spine J.

[6] Lee SH, Derby R, Sul Dg (2011). Efficacy of a new navigable percutaneous disc decompression device (L'DISQ) in patients with herniated nucleus pulposus related to radicular pain. Pain Med.

[7] Wray CC, Easom S, Hoskinson J (1991). Coccydynia. Aetiology and treatment. J Bone Joint Surg Br.

[8] Wright BD (1971). Treatment of intractable coccygodynia by transsacral ammonium chloride injection. Anesth Analg.

[9] Garg B, Ahuja K (2021). Coccydynia-a comprehensive review on etiology, radiological features and management options. J Clin Orthop Trauma.

[10] Sencan S, Yolcu G, Bilim S, Kenis-Coskun O, Gunduz OH (2022). Comparison of treatment outcomes in chronic coccygodynia patients treated with ganglion impar blockade versus caudal epidural steroid injection: a prospective randomized comparison study. Korean J Pain.

[11] Putzier M, Streitparth F, Hartwig T, Perka CF, Hoff EK, Strube P (2013). Can discoblock replace discography for identifying painful degenerated discs?. Eur J Radiol.

[12] DePalma MJ, Lee JE, Peterson L, Wolfer L, Ketchum JM, Derby R (2009). Are outer annular fissures stimulated during diskography the source of diskogenic low-back pain? An analysis of analgesic diskography data. Pain Med.

[13] Liyew WA (2020). Clinical presentations of lumbar disc degeneration and lumbosacral nerve lesions. Int J Rheumatol.

[14] Dellon AL (2015). Pain with sitting related to injury of the posterior femoral cutaneous nerve. Microsurgery.

[15] Li Q, Chen J, Chen Y, Cong X, Chen Z (2016). Chronic sciatic nerve compression induces fibrosis in dorsal root ganglia. Mol Med Rep.

[16] El-Rubaidi OA, Horcajadas-Almansa A, Rodríguez-Rubio D, Galicia-Bulnes JM (2003). Sciatic nerve compression as a complication of the sitting position [Article in Spanish]. Neurocirugia (Astur : Engl Ed).

[17] Son BC, Lee C (2022). Piriformis syndrome (sciatic nerve entrapment) associated with type C sciatic nerve variation: a report of two cases and literature review. Korean J Neurotrauma.

[18] Calisaneller T, Ozdemir O, Karadeli E, Altinors N (2007). Six months post-operative clinical and 24 hour post-operative MRI examinations after nucleoplasty with radiofrequency energy. Acta Neurochir (Wien).

[19] Cuellar VG, Cuellar JM, Vaccaro AR, Carragee EJ, Scuderi GJ (2010). Accelerated degeneration after failed cervical and lumbar nucleoplasty. J Spinal Disord Tech.

[20] Kim JY, Lee KS, Jung SM, Kim YH (2022). Prognostic factors for successful percutaneous disc decompression using the navigable device L’DISQ™ in patients with lumbar discogenic pain. Pain Physician.

